# Exploring the VISTA of microglia: immune checkpoints in CNS inflammation

**DOI:** 10.1007/s00109-020-01968-x

**Published:** 2020-08-27

**Authors:** Malte Borggrewe, Susanne M. Kooistra, Randolph J. Noelle, Bart J. L. Eggen, Jon D. Laman

**Affiliations:** 1 Department of Biomedical Sciences of Cells & Systems, Section Molecular Neurobiology, University Medical Center Groningen, University of Groningen, Groningen, The Netherlands; 2 Department of Microbiology and Immunology, Geisel School of Medicine at Dartmouth, Norris Cotton Cancer Center, Lebanon, NH USA

**Keywords:** Neurodegeneration, Neuroinflammation, Glia cells, Brain disease, Homeostasis

## Abstract

Negative checkpoint regulators (NCR) are intensely pursued as targets to modulate the immune response in cancer and autoimmunity. A large variety of NCR is expressed by central nervous system (CNS)-resident cell types and is associated with CNS homeostasis, interactions with peripheral immunity and CNS inflammation and disease. Immunotherapy blocking NCR affects the CNS as patients can develop neurological issues including encephalitis and multiple sclerosis (MS). How these treatments affect the CNS is incompletely understood, since expression and function of NCR in the CNS are only beginning to be unravelled. V-type immunoglobulin-like suppressor of T cell activation (VISTA) is an NCR that is expressed primarily in the haematopoietic system by myeloid and T cells. VISTA regulates T cell quiescence and activation and has a variety of functions in myeloid cells including efferocytosis, cytokine response and chemotaxis. In the CNS, VISTA is predominantly expressed by microglia and macrophages of the CNS. In this review, we summarize the role of NCR in the CNS during health and disease. We highlight expression of VISTA across cell types and CNS diseases and discuss the function of VISTA in microglia and during CNS ageing, inflammation and neurodegeneration. Understanding the role of VISTA and other NCR in the CNS is important considering the adverse effects of immunotherapy on the CNS, and in view of their therapeutic potential in CNS disease.

## Introduction

Immune checkpoints are critical in maintaining the balance between protective immune responses of appropriate magnitude versus excessive inflammation with undue tissue damage and autoimmune disease. Co-stimulatory and co-inhibitory receptors provide T cells with activating or suppressing signals, respectively, and a disruption of this balance can lead to autoimmunity or prevent specific immune responses. Negative checkpoint regulators (NCR) are receptors that provide co-inhibitory signals to T cells, which lead to inhibition of T cell activation. Targeting immune checkpoints and particularly NCR are intensely pursued as therapeutic targets for cancer and autoimmunity. Blocking NCR enhances anti-tumour immunity, whereas enhancing NCR signalling offers a strategy to alleviate autoimmunity. Studies mainly focus on NCR biology in cancer and peripheral immunity; however, multiple NCR are also expressed by central nervous system (CNS)-resident cell types including neurons, oligodendrocytes, astrocytes and microglia [1]. Expression of most NCR in the CNS is upregulated or induced during inflammation [1]. A subset of cancer patients develops neurological adverse effects after NCR treatment including encephalitis and multiple sclerosis (MS) [1, 2], demonstrating that NCR modulation can affect the CNS. Inhibition of NCR has proven to mount an anti-tumour response in certain types of CNS-associated human tumours [3, 4]. Furthermore, NCR blockade exacerbates CNS autoimmunity such as experimental autoimmune encephalomyelitis (EAE), a mouse model for MS [5]. Detailed clinical studies assessing the effectiveness of modulating NCR in CNS inflammation, ageing and neurodegeneration are lacking.

V-type immunoglobulin domain-containing suppressor of T cell activation (VISTA) is an NCR predominantly expressed by myeloid cells and T cells [6]. In contrast to other NCR, VISTA is expressed on naïve T cells, where it is involved in maintaining T cell quiescence [7] and expression is reduced upon T cell activation. VISTA is also distinct from other NCR because it has a wide diversity of functions in myeloid cells, likely due to its role as a receptor and a ligand. In myeloid cells, VISTA is involved in the uptake of apoptotic cells (efferocytosis) [8], cytokine production [9, 10] and chemotaxis [11]. In the CNS, VISTA is expressed by microglia and to a lesser extent by endothelial cells [12]. Deletion of VISTA exacerbates autoimmunity in mice including EAE [13]. The functions of VISTA in the CNS during health and disease are only beginning to be unravelled. As VISTA can be exploited as a therapeutic target for cancer and autoimmune diseases, it is conceivable that VISTA may offer a novel therapeutic target for CNS inflammation and disease.

In this review, current knowledge of VISTA and other immune checkpoints in the CNS and particularly in microglia are summarized. The potential function of VISTA in microglia and during CNS homeostasis and disease are discussed. Based on published RNA-sequencing studies, we provide novel data on *VISTA* expression in the CNS during health and multiple diseases including neurodegeneration, neuroinflammation, cancer and stroke.

## VISTA expression and function

VISTA (also known as PD-1H [14], DD1a [8], Dies1 [15], Gi24 [16], C10orf54, Vsir, B7H5 and 4632428N05Rik) is an NCR that is expressed in multiple tissues at varying levels. Multiple counterreceptors have been proposed, but not proven beyond doubt. Mainly, immune cells express VISTA on which it acts as both a receptor and a ligand. This dual role and broad expression point towards multiple functions of VISTA, which are discussed in this section.

## VISTA structure and binding partners

VISTA is a transmembrane protein that contains an immunoglobulin variable (IgV)-like fold and shares similarities with B7 family members PD1, PDL1, CD28 and CTLA4 [17]. The extracellular domain of VISTA contains four conserved cysteines that are not present in other B7 family members [17]. Across species, VISTA is highly conserved with 96% identical protein sequence comparing human to other primates (rhesus macaque, cynomolgus monkey, common marmoset) and 77% between human and mouse (unpublished). The *VISTA* gene is located on chromosome 10 within the intronic region of *Cadherin23* (*CDH23*). Of note, regulation of *VISTA* expression seems to be independent of *CDH23* expression [12].

Although the counterreceptor of VISTA remains elusive, multiple candidate binding partners have been proposed: VSIG3/IGSF11 [17, 18], VISTA itself through homophilic interaction [8] and PSGL1 [19]. VSIG3 binds to VISTA in ELISA assays [17, 18], and plate-bound VSIG3 inhibits anti-CD3-induced cytokine secretion by T cells [18]. However, evidence for functional cellular interactions through VISTA and VSIG3 in vitro and particularly in vivo is lacking. A homophilic VISTA interaction between apoptotic cells and macrophages has been suggested to be necessary for facilitating uptake of apoptotic cells [8]. However, this homotypic binding could not be replicated in another study [19]. In this study, PSGL1 was proposed as a binding partner via histidine residues within the extracellular domain of VISTA [19]. Binding of PSGL1 and VISTA leads to inhibition of T cell activation and only occurs at acidic pH in vitro and in vivo (pH 6.0) [19]. Hence, binding of VISTA to PSGL1 selectively occurs in acidic environments, e.g. theoretically provided by tumours and inflammation [19].

It is possible that VISTA has multiple binding partners, but additional evidence and replication studies will be necessary to unequivocally demonstrate functional binding of VISTA to one or more of these potential counterreceptors.

## VISTA expression across tissues and cell types

VISTA mRNA is expressed in multiple organs and tissues including thymus, spleen, heart, kidney, lung, bone marrow and the brain [6]. Predominantly, the hematopoietic compartment expresses VISTA with highest levels in myeloid cells (monocytes, macrophages, dendritic cells), neutrophils, followed by naïve CD4^+^ and CD8^+^ T cells, as well as regulatory Foxp3^+^ T cells [6, 14, 20]. Whereas expression of other NCR is increased upon T cell activation, VISTA is constitutively expressed on resting T cells. VISTA expression in other hematopoietic cell types is detectable but low, including NK cells, thymocytes and plasma cells, whereas no VISTA expression is observed in B cells [6, 14, 20].

Of note, VISTA expression is not restricted to the cell surface, but is also observed in high levels intracellularly in myeloid cells [20]. Here, it colocalizes with markers for early endosomes (EEA-1) and recycling endosomes (Rab-11) [20], suggesting that VISTA is actively recycled and/or has other functions in the cytoplasm.

Several studies demonstrated expression of VISTA in various types of cancer including gastric carcinoma [21], colorectal carcinoma [22, 23], hepatocellular carcinoma [24], ovarian and endometrial cancer [25], prostate cancer [26], pancreatic cancer and melanoma [27]. In some types of cancer, VISTA is expressed by cancer cells themselves, including gastric, ovarian and endometrial tumours [21, 25]. However, VISTA expression is predominantly found on myeloid-derived suppressor cells (MDSC) in the tumour microenvironment [28–31]. In MDSC, VISTA expression is induced by hypoxic tumour environments via hypoxia-inducible factor (HIF)-1a [23]. Moreover, VISTA expression is induced in apoptotic cells as a downstream target of p53 and is required for engulfment by phagocytes [8]. VISTA is also involved in differentiation as reducing VISTA expression using siRNA or miRNA-125b inhibits the differentiation of mouse embryonic stem cells [15, 32] and preadipocytes [33].

## VISTA as a negative checkpoint regulator

Multiple studies have demonstrated that VISTA inhibits T cell activation and therefore functions as an NCR. VISTA-Ig fusion protein or VISTA-overexpressing A20 cells both reduce proliferation and cytokine production (Il2 and Ifng) in ovalbumin (OVA) or anti-CD3-stimulated T cells in vitro [6]. Furthermore, blocking VISTA in mice using an antagonistic anti-VISTA antibody (clone 13F3) increases T cell proliferation in response to OVA and exacerbates the development of EAE, a model for MS [6]. Concomitantly, targeting VISTA on T cells in mice using an agonistic anti-VISTA antibody (clone MH5A or 8G8) protects mice from graft-versus-host disease GvHD [14], hepatitis [34], lupus [7, 35], psoriasis [7] and arthritis [7]. This protection from GvHD is independent of host cells [14] and is due to engagement of VISTA on donor T cells, inhibiting their activation [7, 36].

In addition to inhibition of T cell activation, VISTA is also involved in T cell differentiation and expansion. In GvHD, for example, activation of VISTA on donor T cells expands regulatory T cells (Tregs) [14]. Concordantly, T cells in generic VISTA knockout (KO) mice exhibit a reduced ability to form iTregs [37]. The generation of natural Tregs, however, is not impaired [37]. The iTregs of generic VISTA KO mice are more prone to conversion into T helper 17 (Th17) and Th1 cells during inflammation compared to wild-type iTregs [37]. This overall reduction in iTreg formation and induction of Th1 and Th17 cells supports the notion of a more reactive T cell compartment in VISTA KO mice. It is likely that this reactivity is caused by both intrinsic effects of VISTA deficiency in T cells, and indirect effects of an altered cytokine profile and depletion of VISTA in other cell types (e.g. dendritic cells, DC). Consistent with this argument, DC in VISTA KO mice produce more Il23, leading to augmented Il17a production by Th17 and γδ T cells, resulting in the exacerbation of psoriasiform plaques in mice induced by imiquimod [38].

In contrast to other NCR which are expressed upon T cell activation, VISTA is constitutively expressed on resting T cells, suggesting distinct functionalities. Underscoring this non-redundant role of VISTA, double KO of VISTA and PD1 significantly increases T cell responses to foreign antigens and exacerbates EAE compared with VISTA or PD1 single KO mice [39]. Detailed analysis of the T cell compartment in VISTA KO mice using single-cell transcriptomic and epigenetic approaches demonstrates that VISTA is crucial for maintaining naïve T cell quiescence [7]. Therefore, VISTA regulates T cell tolerance before activation occurs, whereas other NCR such as CTLA4 and PD1 only act after T cell activation to inhibit priming and effector functions. VISTA is the first known NCR that acts at such an early stage in the T cell activation cascade and hence offers a novel, non-redundant target for therapeutic interventions [7].

## VISTA in myeloid cell biology

VISTA was initially discovered as an NCR, but since then, a role for VISTA in a variety of other functions in myeloid cells has been proposed, including cytokine response, chemotaxis and efferocytosis.

In multiple mouse models of inflammation, VISTA KO is associated with an increase in pro-inflammatory cytokines [38, 40–42]. These cytokines derive from T cells and myeloid cells. In a psoriasis mouse model, for example, VISTA KO enhances the production of Il23 by DCs [38]. Surprisingly, overexpression of VISTA in human monocytes in vitro leads to spontaneous cytokine production (TNF, IL1β) on mRNA [9] and protein level [10]. It is unclear whether these opposing findings are due to differences between species (human versus mouse), or because of distinct approaches of studying VISTA (KO versus overexpression). Nonetheless, these studies demonstrate that VISTA is involved in the cytokine response of myeloid cells.

Emerging evidence suggests that VISTA is involved in chemotaxis and migration through direct and indirect signalling in myeloid cells. In VISTA KO mice, elevated levels of inflammatory cytokines and chemokines such as Ccl2 (MCP1) are observed in the lung, which is associated with the development of experimental asthma [41]. Ccl2 is a chemoattractant for monocytes, and thus, VISTA may indirectly regulate the recruitment of monocytes. VISTA also directly regulates monocyte chemotaxis, since blocking VISTA on monocytes using an antagonistic antibody (clone 13F3) enhances their migration ability [11]. Concordantly, expression of the Ccl2 receptor Ccr2 was increased in 13F3-treated mice [11].

In macrophages, expression of VISTA is required for the engulfment and uptake of apoptotic cells in vivo and in vitro [8, 43]. VISTA is upregulated in a p53-dependent manner in apoptotic cells and a homophilic interaction with VISTA on macrophages facilitates efferocytosis [8]. A lack of VISTA on either phagocytes or apoptotic cells impairs dead cell clearance [8]. However, as mentioned, a homophilic interaction of VISTA could not be replicated to date [19]. Concordantly, blocking VISTA on macrophages using a neutralizing antibody also reduces the uptake of neutrophils in vitro [43].

Many of the presented experiments are based on a generic VISTA KO mouse model or systemically administered VISTA-modulating antibodies. Therefore, it cannot be excluded that some of the observed changes in myeloid cells are due to a lack of VISTA on other cell types as opposed to a cell-intrinsic role of VISTA. However, most studies additionally used cell-specific in vitro assays to verify their results, suggesting a cell-intrinsic function. Using conditional depletion of VISTA in a cell-type-specific manner will be important to further dissect the function of VISTA in myeloid cells in vivo.

In summary, VISTA functions beyond being an NCR and is involved in multiple aspects of the innate immune response of myeloid cells.

## Dual role of VISTA as receptor and ligand

VISTA has a large spectrum of expression and functions across multiple tissues and cell types. This diverse function and expression may in part be attributed to the dual role of VISTA as a receptor and a ligand. However, despite major efforts and hampered by the lack of optimal functional assays, whether VISTA has a single or multiple ligands and receptors remains in need of further clarification and verification.

Regarding the function of VISTA as an NCR, both ligand and receptor activities on APC and T cells can lead to T cell inhibition. VISTA-Ig fusion proteins and VISTA-overexpressing A20 cells both reduce proliferation of anti-CD3-stimulated T cells [6, 44]. Therefore, VISTA expressed on APC can act as a ligand, and upon binding to a counterreceptor on T cells, this leads to T cell inhibition. Conversely, engaging VISTA expressed on naïve T cells can also inhibit T cell activation, which has been shown in the context of hepatitis [34] and GvHD in mouse models [7, 14]. As mentioned, treatment of mice with agonistic anti-VISTA antibody (clone MH5A) activating VISTA signalling protects mice against GvHD [14]. Passive transfer of wild-type T cells into VISTA KO mice and subsequent anti-VISTA treatment also reduced GvHD, demonstrating that host cells are not involved in this protective effect [14]. Thus, VISTA as a receptor on T cells and as a ligand on T cells as well as APC inhibits T cell activation and thereby exerts its role as an NCR.

In myeloid cells, VISTA also acts as a receptor thereby exerting functions beyond inhibition of T cell activation. Overexpression of VISTA in human monocytes/macrophages leads to spontaneous inflammatory cytokine secretion, which is abrogated after deleting the cytoplasmic domain [10]. Although the cytoplasmic domain of VISTA does not contain any immunoreceptor tyrosine-based signalling motifs, multiple casein kinase 2 and phosphokinase C phosphorylation sites are present [17]. These data demonstrate that engagement of VISTA on myeloid cells results in downstream cellular signalling through the cytoplasmic tail, which has functional ramifications for the cell such as cytokine production [10].

This dual role of VISTA as a receptor and ligand has important consequences for studying its function and the therapeutic potential of anti-VISTA antibodies. The effect of VISTA KO and VISTA-targeted treatment must be studied for individual cell types and with regard to VISTAs broad functions.

## VISTA in the CNS

Expression and function of NCR in peripheral immunity especially during cancer and autoimmunity are extensively studied and are beginning to be understood. In contrast, the physiological and pathological functions of NCR in the CNS are in its infancy, although NCR are likely involved in a variety of CNS functions including communication with peripheral immune cells. The extensive functional diversity of VISTA in myeloid cells points towards functional relevance for VISTA in CNS-resident myeloid cells: microglia. In this part, expression and function of NCR and particularly VISTA in microglia are discussed.

## Immune checkpoints in the CNS

In the CNS, various co-stimulatory and co-inhibitory receptors are expressed by CNS-resident cells and during disease also by infiltrating immune cells. In this review, we focus on inhibitory immune checkpoints and particularly VISTA expression and function in CNS-resident cells.

**Fig. 1:**
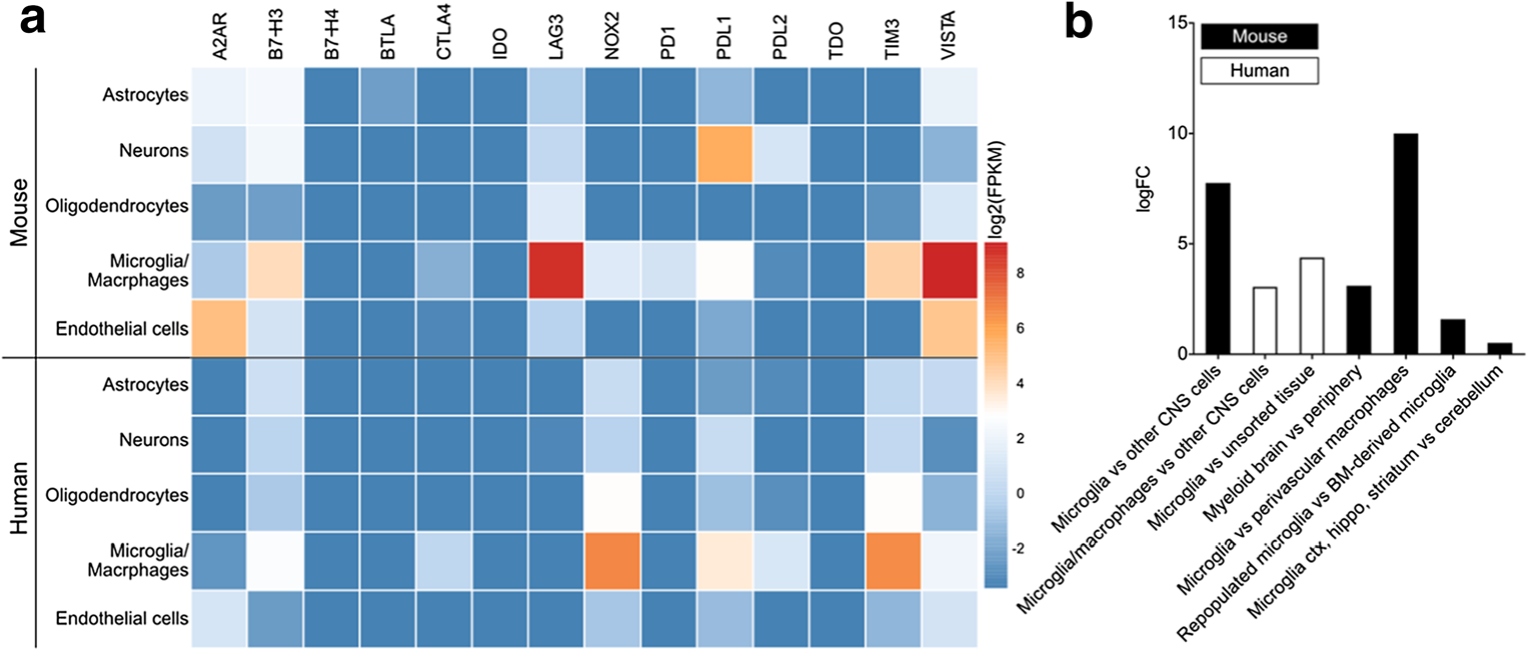
Expression of VISTA and other inhibitory immune checkpoints in mouse and human CNS during homeostasis. **a** Heatmap illustrates mRNA levels as log2(FPKM) in different types of CNS cells, derived from published mRNA sequencing data [45, 46]. **b** Log fold change (logFC) of *VISTA* expression in microglia compared with other CNS cells, myeloid cells, bone marrow-derived microglia and in different CNS regions (Table 1). FPKM = fragments per kilobase million, logFC = log2FoldChange, BM = bone marrow

Multiple inhibitory immune checkpoints are expressed by mouse and human CNS-resident cells at least on mRNA level with varying abundancies including A2AR, B7-H3, BTLA, CTLA4, LAG3, NOX2, PD1, PDL1, PDL2, TIM3 and VISTA (Fig. 1). Every major CNS cell type (neurons, oligodendrocytes, microglia, astrocytes and endothelial cells) expresses inhibitory immune checkpoints, but microglia express the largest diversity (Fig. 1). Expression of many of these inhibitory immune checkpoints is induced or upregulated during inflammatory conditions including PD1, PDL1, PDL2 and TIM3 [1].

Function and in-depth expression dynamics of the majority of immune checkpoints have not been studied in detail in the CNS. The best studied NCR in the CNS is PDL1 (also known as CD274 and B7-H1), which is predominantly expressed by microglia and neurons (Fig. 1). During inflammation, PDL1 expression is induced in astrocytes, oligodendrocytes [67] and endothelial cells [68] and upregulated in microglia [12] and neurons. Upregulation or induction of PDL1 in microglia and astrocytes during inflammation may limit CNS inflammation and pathology by inhibition of T cell activation [69]. In EAE, for example, responses of infiltrating PD1-expressing T cells are suppressed by microglia PDL1 expression [70]. Conversely, deletion of PDL1 or PDL2 in mice reduces the infarct volume after middle cerebral artery occlusion, due to reduction of immune-activated microglia and infiltrating peripheral immune cells [71]. Hence, it is possible that expression of PDL1 has different functional consequences for different cell types (T cells vs myeloid cells). In PDL1-deficient mice, an increase in PD1 and PDL2 expression was detected [71], suggesting a compensatory mechanism. Therefore, genetic depletion of one NCR can likely be balanced by upregulation of functionally similar NCR.

TIM3 is a co-inhibitory receptor that suppresses T cell activation. In microglia, TIM3 regulates inflammatory responses such as iNOS production after exposure to glioma-conditioned medium [72], suggesting that NCR have intrinsic functions in microglia biology. In melanoma brain metastases, microglia are the principal IDO-expressing cell type compared with infiltrating immune cells [73]. IDO is an immunomodulatory enzyme that facilitates conversion of tryptophan to kynurenine, resulting in antimicrobial and immunosuppressive environments [74]. This high expression of IDO indicates that microglia are potent immunomodulatory cells especially during CNS diseases that include immune cell infiltration, such as MS.

## Expression of VISTA in the CNS

*VISTA* mRNA is expressed in whole brain tissue, but much lower compared with thymus, spleen and lung [6]. To our knowledge, there is only one study to date that focused on VISTA expression in the CNS [12]. In that study, we demonstrate that in the mouse and human CNS, VISTA is predominantly expressed by microglia, which are the parenchyma resident myeloid cells [12] (also shown in Fig. 1a, b). During mouse and human development, microglia *VISTA* expression increases gradually with highest expression in adult microglia [12, 75, 76]. *VISTA* expression levels in microglia are comparable with well-established microglia markers such as CX3CR1, TMEM119, P2RY12 and ITGAM (CD11B) [12, 47]. CNS myeloid cells (microglia and brain-border macrophages) express higher levels of *VISTA* than peripheral myeloid cells, and *VISTA* expression is higher in microglia compared with perivascular macrophages (Fig. 1b). Interestingly, after diphtheria toxin-induced ablation of microglia expressing diphtheria toxin receptor, *VISTA* expression is higher in repopulated microglia than in bone marrow-derived microglia (Fig. 1b). Together, these results suggest that microglia VISTA expression is higher compared with peripheral myeloid cells, which express the highest levels of VISTA among peripheral immune cells [6, 14, 20].

ATAC-seq data suggests that VISTA expression is regulated by SPI1/PU.1 [12], a transcription factor essential for microglia and myeloid cell biology. In mouse spinal cord and brain, more than 95% of microglia (Cd11b^pos^Cd45^int^) express VISTA, whereas only few Cd11b^neg^Cd45^neg^ cells express VISTA [12]. These VISTA positive non-microglia cells are most likely endothelial cells, since endothelial cells express low but detectable mRNA levels of *VISTA* [12]. Furthermore, blood vessels are positive for VISTA in immunohistochemical staining of mouse and human brain, which underscores endothelial VISTA expression. RNA sequencing data also suggests expression of *VISTA* by astrocytes; however, protein expression was not detectable by immunohistochemical staining [12]. Expression dynamics and potential function of VISTA during inflammation, ageing and CNS diseases are further discussed below.

## Potential functions of VISTA in microglia

**Fig. 2:**
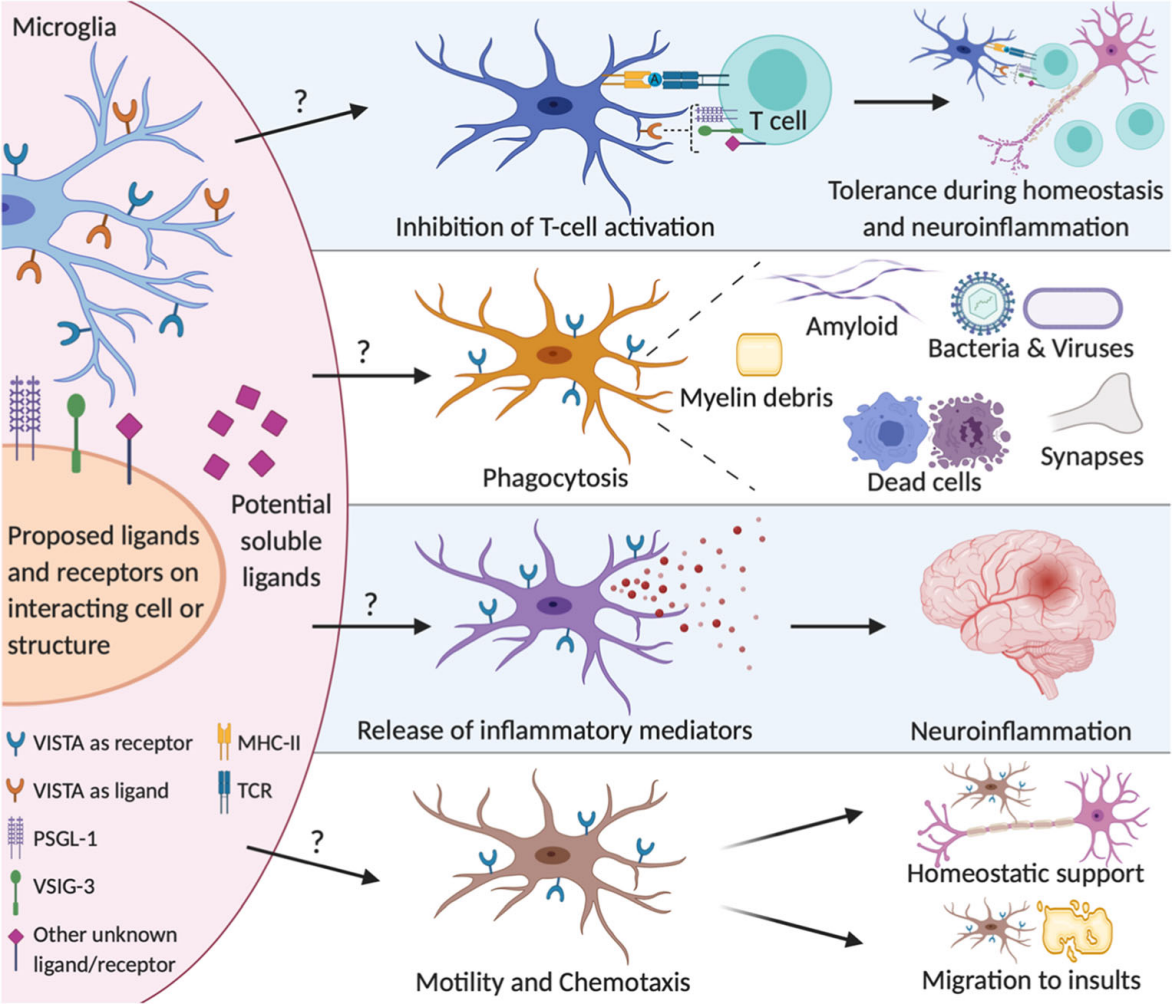
Potential functions of VISTA in microglia and the effect on CNS homeostasis and disease. VISTA expressed on microglia may act as a receptor and a ligand, binding to proposed and unknown ligands/receptors. Based on VISTA function in myeloid cells, VISTA may be involved in microglia functions such as antigen-presentation, phagocytosis, release of inflammatory mediators and motility and chemotaxis. These microglia functions are important for maintaining CNS homeostasis including synaptic pruning, removal of metabolic waste and cell debris and immune tolerance. Furthermore, these potential functions of VISTA in microglia are essential during CNS disease, in which microglia are responsible for antigen presentation, defence against pathogens, protective versus destructive neuroinflammation and for tissue regeneration

Microglia are myeloid cells of the CNS and possess similar functions as tissue macrophages such as antigen presentation, phagocytosis, respiratory burst and release of cytokines and chemokines [77]. As opposed to other tissue-macrophage subsets, microglia also exhibit a range of CNS-specific functions including synaptic pruning, and the release of neurotrophic as well as neurotoxic factors [77]. During homeostasis, microglia are constantly scanning their environment and are highly sensitive and responsive towards any perturbations [77]. Hence, the notion of resting microglia has become obsolete. Despite major efforts, defining microglia functionality in the M1-M2 continuum has been unproductive and contentious [78]. The function of VISTA in microglia and the CNS is unknown. In this paragraph, potential functions of VISTA in microglia are discussed based on known functions of VISTA in other myeloid cells (Fig. 2).

Since microglia are capable of presenting antigens and expressing other NCR, it is conceivable that VISTA as a ligand also acts as an NCR in microglia, where it binds to a counterreceptor on T cells leading to inhibition of T cell activation (Fig. 2). VISTA functioning as an NCR in microglia might be of particular relevance for CNS-peripheral immunity interactions (discussed below), which predominantly occur during immune cell infiltration in CNS diseases such as MS. In view of VISTA ligand functions as an NCR, it is intriguing that microglia express VISTA at such high levels during steady state, since peripheral immune cells including T cells are sparse in healthy brain parenchyma. Therefore, it is likely that in addition to inhibiting T cell activation, VISTA has a function in microglia as a receptor.

In monocytes/macrophages, VISTA as a receptor is involved in efferocytosis [8], cytokine production [10] and chemotaxis [11]. Since microglia are functionally closely related to monocytes/macrophages, it is possible that one or more of these functions is also regulated by VISTA in microglia (Fig. 2). Particularly, the involvement of VISTA in efferocytosis would be highly relevant for microglia. Microglia are responsible for clearing cellular debris in the brain, especially during development and disease [77] (Fig. 2). Furthermore, microglia are involved in synaptic pruning (synaptophagy) [77], a specific form of phagocytosis to eliminate viable synapses, which is required for learning and memory. VISTA as a receptor might be involved in these processes, in view of its role in phagocytosis in macrophages [8].

Microglia are highly capable of producing pro and anti-inflammatory cytokines and chemokines upon receiving a wide variety of stimuli [77]. The intracellular pathways leading to the production of these signalling molecules are mostly conserved between microglia and other tissue macrophages. Since overexpression of VISTA in vitro leads to spontaneous cytokine secretion (TNF, IL1β) in human monocytes [10], and knockout of VISTA is associated with an altered cytokine/chemokine profile (Ccl2, Il23) [38, 40–42], VISTA may also be involved in microglia cytokine/chemokine production.

VISTA as a receptor is not only involved in the production, but also in the response to chemokines. Blocking VISTA in mice leads to enhanced migratory capacity of monocytes in response to Ccl2 in vitro [11]. Ccl2 in the CNS is produced by astrocytes, microglia, endothelial cells [79] and can be produced by neurons during stress such as impairment of oxidative metabolism [80]. Microglia express Ccr2 and respond to Ccl2 by migrating and producing cytokines [79]. This response can be both beneficial and detrimental as it leads to clearance of debris, but also contributes to neuroinflammation by production of pro-inflammatory cytokines [79, 80]. VISTA could be involved in the microglia response/migration to Ccl2, which would have ramifications particularly during CNS diseases where Ccl2 production is increased such as MS, traumatic brain injury and stroke [79] (Fig. 2).

## Roles of VISTA and other NCR in CNS-peripheral immunity interactions

NCR are pivotal signalling molecules that aid in balancing immune responses to limit autoimmunity while maintaining an effective immune response. Therefore, it is important to discuss the role of NCR and VISTA with regard to their direct signalling capacity through cell-cell interactions, in this case, the interaction between CNS and peripheral immunity. There are two main types of CNS-peripheral immunity interactions: indirect (e.g. through cytokines and other secreting signalling molecules) and direct (cell-cell contact through receptors) [81]. We will focus on direct interactions of glia and endothelial cells with the peripheral immunity via NCR and VISTA.

The initial contact of peripheral immune cells with the CNS is via endothelial cells, which directly interact with immune cells. Endothelial cells can regulate the trans-migration of the peripheral immune cells into the CNS, which is of particular importance during CNS diseases such as MS [82]. Blocking the adhesion of immune cells to the endothelium by natalizumab blocking VLA4 emerged as an effective therapy to limit neuroinflammation [83]. Endothelial cells are capable of presenting antigens through MHC-II and can facilitate the trans-migration of T cells into the brain parenchyma [84]. As capable APC, endothelial cells also express a range of NCR (Fig. 1) such as PDL1 and PDL2 which suppress T cell responses in vitro [85] and may inhibit T cell trans-migration. VISTA as a ligand expressed by endothelial cells may provide inhibitory signals to passing T cells as well, thereby fine-tuning T cell reactivity in the CNS, which is of particular importance in CNS diseases with immune cell infiltration (e.g. MS). The function of VISTA in endothelial cells has not been studied to date but should be investigated particularly with regard to peripheral immune cell infiltration into the CNS and antigen presenting capability of the endothelium.

Other CNS cell types that can actively communicate with peripheral immune cells via direct contact are astrocytes and microglia. Both cell types can express MHC-II (induced/upregulated during inflammation notably by interferons) and are capable of presenting antigens to T cells [86], and both cell types express NCR, as previously mentioned (Fig. 1). Microglia PDL1 expression regulates T cell (re)activation in the CNS during EAE [70, 87]. In the transgenic APP/PS1 (APPswe/PS1dE9) mouse model for Alzheimer’s disease, depletion of microglia using a Csf1r inhibitor (PLX5622) results in an increase in parenchymal T cell numbers and a reduction of anti-inflammatory cytokines [88]. It is thus conceivable that microglia provide inhibitory signals to T cells, which is essential to limit T cell (re)activation in the CNS. Functional evidence on whether VISTA expressed by microglia has co-inhibitory effects on T cell activation is lacking; however, based on extensive characterisation of VISTA NCR functions in other myeloid cells, it is highly likely that blocking or depleting VISTA on microglia will enhance T cell (re)activation in the brain.

## VISTA in CNS diseases and aging

**Fig. 3:**
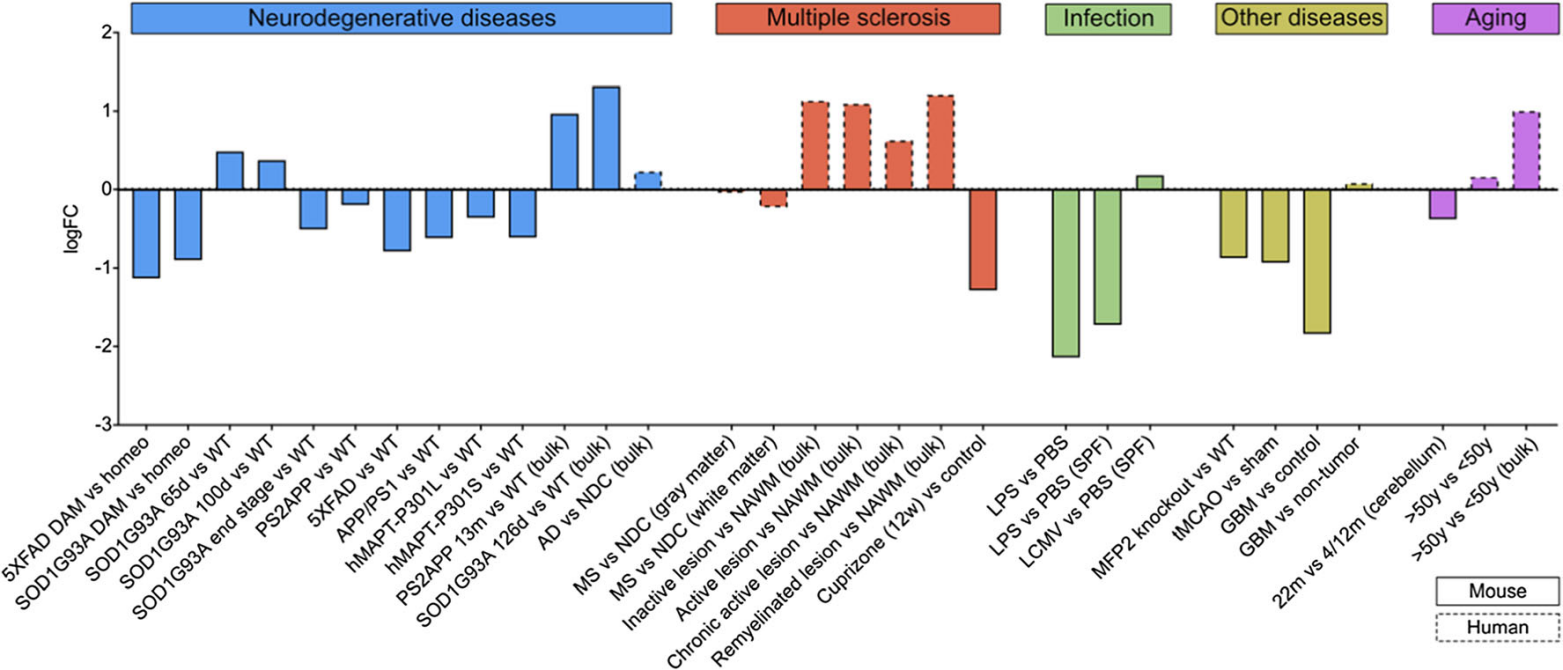
VISTA expression in CNS diseases and aging. Log fold change (logFC) of *VISTA* expression in microglia or bulk CNS tissue during disease compared with control (Table 1). DAM = disease-associated microglia, Homeo = homeostatic microglia, WT = wild-type, AD = Alzheimer’s disease, NDC = non-demented control, MS = multiple sclerosis, NAWM = normal-appearing white matter, LPS = lipopolysaccharide, PBS = phosphate-buffered saline, LCMV = lymphocytic choriomeningitis virus, SPF = specific-pathogen free, MFP2 = multifunctional protein-2, tMCAO = transient middle cerebral artery occlusion, GBM = glioblastoma

Microglia are involved in CNS diseases by mounting inflammatory responses, assisting in clearance of waste and defending the CNS from intruder pathogens and toxic compounds. As described above, expression of most NCR is induced/upregulated in microglia and other CNS cell types during inflammation and CNS disease, e.g. PDL1 [12]. VISTA expression, however, is decreased in microglia during CNS inflammation and disease [12]. To expand on these observations, we analysed *VISTA* expression with focus on microglia in published mRNA sequencing datasets of multiple CNS diseases and respective animal models including neurodegenerative diseases (NDD), MS, infection, stroke, glioblastoma (GBM) and aging. This analysis is summarized in Fig. 3 and discussed in more detail below.

## Neurodegenerative diseases

NDD, including AD, frontotemporal dementia (FTD), Parkinson’s disease (PD) and amyotrophic lateral sclerosis (ALS), are progressive degenerative diseases of the CNS. Hallmarks of NDD are the loss of neurons and neuroinflammation. Microglia are the major source of neuroinflammation in NDD and significantly contribute to development and progression of these diseases [89, 90]. However, microglia also phagocytose cellular debris and plaques that are formed in many NDD, thereby facilitating clearance of waste. Hence, microglia appear to have both beneficial and detrimental functions in NDD.

In the AD mouse model 5XFAD and ALS model SOD1G93A, microglia downregulate expression of homeostatic genes, while upregulating genes involved in immune activation and phagocytosis [52]. This NDD-associated microglia phenotype is also called disease-associated microglia (DAM or MGnD) [52, 91, 92]. DAM microglia in both AD and ALS models exhibit 2-fold reduced *VISTA* expression (Fig. 3, Table 1). The decrease in microglia *VISTA* expression is consistent across multiple AD mouse models including 5XFAD, APP/PS1 and PS2APP (Fig. 3, Table 1). In spinal cord microglia from ALS SOD1G93A mice, *VISTA* expression is slightly upregulated in early stages, but decreased during the end stage of disease (Fig. 3, Table 1). In tau mouse models that carry P301L or P301S mutations associated with FTD and PD, *VISTA* expression in microglia is also reduced (Fig. 3, Table 1). Collectively, these data point towards *VISTA* being regulated in microglia similar to homeostatic markers, which are also decreased during microglia activation and in NDD [90].

**Table 1:** *VISTA* expression in microglia, CNS diseases and ageing

	Description	Species	Tissue	Cell subset	Condition	logFC	padj	Reference	
Microglia in healthy CNS	Microglia vs other CNS cells	Mouse	Cortex	Cd45+	Control	7.75	0.006	[46]	a
	Myeloid vs other CNS cells	Human	Temporal cortex	CD45+	Control	3.05	0.001	[45]	^a^
	Microglia vs unsorted tissue	Human	Cortex	CD11B+CD45int	Control	4.06	0.000	[47]	
	Myeloid brain vs periphery	Mouse	Brain, peripheral tissues	Cd11b+Cd45int	Control	3.09	0.015	[48]	^a^
	Microglia vs perivascular macrophages	Mouse	Somatosensory cortex, CA1 hippocampus	Myeloid (scRNAseq)	Control	10.00	1.000	[49]	^a^
	Repopulated microglia vs BM-derived microglia	Mouse	Brain	Cd11b+Cd45int	Control	1.58	0.001	[50]	^a^
	Microglia ctx, hippo, striatum vs cerebellum	Mouse	Cerebellum, cortex, hippocampus, striatum	Cd11b+	Control	0.51	0.000	[51]	^a^
Neurodegenerative diseases	5XFAD DAM vs homeo	Mouse	Brain	Cd45+	5XFAD	− 1.13	NA	[52]	
	SOD1G93A DAM vs homeo	Mouse	Spinal cord	Cd45+	SOD1G93A	− 0.90	NA	[52]	
	SOD1G93A 65d vs WT	Mouse	Spinal cord	Cd11b+	SOD1G93A	0.49	NA	[53]	
	SOD1G93A 100d vs WT	Mouse	Spinal cord	Cd11b+	SOD1G93A	0.38	NA	[53]	
	SOD1G93A end stage vs WT	Mouse	Spinal cord	Cd11b+	SOD1G93A	− 0.51	NA	[53]	
	PS2APP vs WT	Mouse	Cortex	Cx3cr1::Gfp+	PS2APP	− 0.20	0.496	[54]	^a^
	5XFAD vs WT	Mouse	Brain	Cd11b+Cd45int	5XFAD	− 0.79	0.078	[55]	^a^
	APP/PS1 vs WT	Mouse	Cortex	Cd11b+Cd45int	APP/PS1	− 0.62	0.000	[56]	^a^
	hMAPT-P301L vs WT	Mouse	Hippocampus	Cd11b+	hMAPT-p301L	− 0.36	0.608	[54]	^a^
	hMAPT-P301S vs WT	Mouse	Hippocampus	Cd11b+	hMAPT-p301S	− 0.61	0.124	[54]	^a^
	PS2APP 13m vs WT (bulk)	Mouse	Cortex	Bulk tissue	PS2APP	0.97	0.000	[57]	^a^
	SOD1G93A 126d vs WT (bulk)	Mouse	Spinal cord	Bulk tissue	SOD1G93A	1.32	0.007	[58]	^a^
	AD vs NDC (bulk)	Human	Fusiform gyrus	Bulk tissue	AD	0.23	0.150	[54]	^a^
Multiple sclerosis	MS vs NDC (gray matter)	Human	Gray matter	CD15-CD11B+	MS	− 0.04	NA	[59]	
	MS vs NDC (white matter)	Human	White matter	CD15-CD11B+	MS	− 0.23	NA	[59]	
	Inactive lesion vs NAWM (bulk)	Human	White matter	Bulk tissue	MS	1.14	NA	[60]	
	Active lesion vs NAWM (bulk)	Human	White matter	Bulk tissue	MS	1.10	NA	[60]	
	Chronic active lesion vs NAWM (bulk)	Human	White matter	Bulk tissue	MS	0.63	NA	[60]	
	Remyelinated lesion vs NAWM (bulk)	Human	White matter	Bulk tissue	MS	1.22	NA	[60]	
	Cuprizone (12w) vs control	Mouse	Brain	Cd11b+Cd45int	Cuprizone	− 1.28	0.424	[61]	^a^
Infection	LPS vs PBS	Mouse	Cortex	Cd11b+	LPS	− 2.14	0.000	[57]	^a^
	LPS vs PBS (SPF)	Mouse	Brain	Cd11b+Cd45int	SPF, LPS	− 1.72	0.363	[62]	^a^
	LCMV vs PBS (SPF)	Mouse	Brain	Cd11b+Cd45int	SPF, LCMV	0.19	0.918	[62]	^a^
Other diseases	MFP2 knockout vs WT	Mouse	Brain	Cd11b+Cd45int	MFP2 knockout	− 0.87	0.091	[63]	^a^
	tMCAO vs sham	Mouse	Cortex	Cd11b+Cd45int	tMCAO	− 0.93	0.414	[64]	^a^
	GBM vs control	Mouse	Brain, tumour	Cd11b+	Glioma	− 1.84	0.002	[65]	^a^
	GBM vs non-tumour	Human	Brain, tumour	Microglia (scRNAseq)	Glioma	0.08	NA	[66]	
Ageing	22m vs 4/12m (cerebellum)	Mouse	Cerebellum	Cd11b+	Control	− 0.38	0.161	[51]	^a^
	> 50 years vs < 50 years	Human	Cortex	CD11B+CD45int	Control	0.16	NA	[47]	
	> 50 years vs < 50 years (bulk)	Human	Cortex	CD11B+CD45int	Control	1.00	NA	[47]	

^a^Data from myeloid brain expression meta-analysis [54]

*BM* bone marrow, *ctx* cortex, *hippo* hippocampus, *DAM* disease-associated microglia, *Homeo* homeostatic microglia, *WT* wild-type, *AD* Alzheimer’s disease, *MS* multiple sclerosis, *tMCAO* transient middle cerebral artery occlusion, *SPF* specific pathogen free, *LPS* lipopolysaccharide, *LCMV* lymphocytic choriomeningitis virus, *MFP2* multifunctional protein-2, *GBM* glioblastoma, *KO* knockout, *NA* not available

Although the function of VISTA in microglia remains unknown, VISTA knockout in myeloid cells leads to decreased phagocytosis and elevated production of cytokines [8, 38, 41]. Therefore, reduction in microglia *VISTA* expression during NDD could have detrimental effects, as it might enhance neuroinflammation while inhibiting the clearance of cell debris and waste.

Surprisingly, *VISTA* gene expression in bulk tissue from AD and ALS mice and in post-mortem human AD tissue is elevated (Fig. 3, Table 1). Endothelial cells express low levels of *VISTA* in non-diseased conditions, but it is possible that expression is upregulated during NDD. Furthermore, *VISTA* expression might be induced in other CNS cell types in NDD, which do not express VISTA under homeostatic conditions.

Together, *VISTA* expression by microglia is consistently decreased in multiple models of NDD, which could have detrimental effects. However, bulk tissue gene expression data indicates that other CNS cell types upregulate or induce *VISTA* expression in these conditions, warranting further investigation.

## Multiple sclerosis

MS is a chronic demyelinating autoimmune disease of the CNS characterised by formation of demyelinated areas or lesions that contain peripheral immune cell infiltrates dominated by macrophages. The heterogeneous nature of MS manifests in highly individual disease courses, a diversity of clinical symptoms and the emergence of different types of lesions. These lesions can be staged based on degree of demyelination and inflammation [93, 94]. Based on studies using a wide variety of EAE models, microglia are believed to be beneficial during initial disease responses in order to resolve inflammation and promote tissue regeneration [95]. Later during the disease, microglia may contribute to chronic neuroinflammation and neurodegeneration.

Microglia *VISTA* expression is reduced during all stages of actively induced disease by MOG_35-55_ in CFA [12], and VISTA knockout exacerbates EAE in a spontaneous TCR-transgenic [13]. Cuprizone-feeding in mice is a model in which chemical-induced death of oligodendrocytes leads to demyelination and remyelination, and microglia immune-activation in the absence of peripheral immune cell infiltrates. *VISTA* expression in microglia is also reduced in this MS mouse model (Fig. 3, Table 1). Furthermore, VISTA expression is decreased in chronic active MS lesions [12], jointly suggesting a role for VISTA in MS.

Most MS lesions occur in white matter (WM); however, grey matter (GM) lesions are frequent and are a hallmark of MS. In MS WM, microglia *VISTA* expression is slightly decreased compared with WM of non-demented controls (NDC), whereas no difference is evident in MS GM (Fig. 3, Table 1).

One hallmark of MS lesions and EAE is the infiltration of peripheral immune cells including macrophages and lymphocytes. More recently, neutrophils were also associated with lesion formation and MS pathology [96]. A loss or reduction of VISTA expression on microglia in MS/EAE may boost (re)activation of infiltrating T cells in lesions, thereby exacerbating inflammation and tissue damage. Moreover, reduced VISTA levels in microglia and infiltrating monocytes may impair their phagocytic ability, which is important for clearance of cellular and myelin debris early during the disease [95]. The role of VISTA in microglia in MS/EAE might depend on the stage of disease and the type of MS lesion, including the lesion microenvironment and how microglia respond to these environmental cues.

Microglia-specific gene expression in different types of MS lesions has not been studied yet; however, data on bulk tissue from different lesions is available. Here, *VISTA* expression is upregulated in all investigated types of lesions including inactive, active, chronic active and remyelinated (Fig. 3, Table 1). It remains conceivable that microglia *VISTA* expression is reduced, but this cannot be detected in bulk tissue when other cell types upregulate/induce *VISTA* expression. As discussed above, although endothelial VISTA expression is low under homeostatic conditions, it might be upregulated during non-homeostatic conditions. Furthermore, other CNS cell types may induce VISTA expression, explaining elevated *VISTA* levels in bulk tissue. *VISTA* is likely also expressed by infiltrating immune cell subsets including neutrophils, lymphocytes and myeloid cells in MS lesions. VISTA is upregulated at least in myeloid cells under inflammatory conditions [10], which would also explain elevated *VISTA* levels in MS lesions. It will be important to assess cell type-specific VISTA expression in different types of MS lesions to dissect the role of VISTA in microglia and other cell types during MS development and progression.

## Infection

Microglia express a range of pattern-recognition receptors such as toll-like receptors (TLR), C-type lectin receptors and NOD-like receptors, which allows them to sense and respond to pathogen-associated and damage-associated molecular patterns [97]. In the CNS, microglia are the major cell type capable of monitoring and defending the tissue from intruders including bacteria and viruses. Upon response towards microbial compounds such as LPS (TLR4), polyI:C (TLR3), β-glucan (Dectin-1, TLR2/6), Pam3CSK4 (TLR1/2), *VISTA* expression decreases in mouse and rhesus macaque microglia in vitro by 50–70% [12]. A similar decrease is observed in mouse microglia 3/6/24 h after intraperitoneal LPS injection [12] (Fig. 3, Table 1). Although VISTA expression is reduced after LPS injection, it is not altered during infection with lymphocytic choriomeningitis virus (LCMV) by intracerebral inoculation (Fig. 3, Table 1).

The lack of studies on infections in relation to VISTA biology in the CNS underscores that this important topic remains largely unexplored.

## Other CNS diseases and aging

By contributing to neuroinflammatory mechanisms, microglia are also involved in a range of other neurological diseases including stroke, cancer and more. *VISTA* expression by microglia is reduced in almost all CNS disease conditions including multifunctional protein-2 (MFP2) knockout mice (Fig. 3, Table 1). MFP2 defects in humans usually lead to severe developmental pathologies including neonatal hypotonia, seizures, psychomotor retardation and brain malformations [98]. In mice, MFP2 knockout leads to Purkinje cell degeneration and neuroinflammation [98].

During transient middle-cerebral artery occlusion (tMCAO), which leads to stroke in mice, microglia *VISTA* expression is reduced 2-fold (Fig. 3, Table 1). Although inhibition of microglia activation during stroke leads to beneficial outcomes, microglia activation is also necessary to counteract neuronal death and enhance neurogenesis [99].

Microglia and macrophages are part of the tumour environment in GBM and promote tumour progression by producing anti-inflammatory cytokines, immunosuppressive molecules and angiogenic factors [100]. Although microglia acquire a more immune-silencing phenotype characterized by secretion of anti-inflammatory cytokines and an upregulation of NCR, *VISTA* expression is reduced in mouse microglia and unaltered in human microglia associated with GBM (Fig. 3, Table 1). A decrease in *VISTA* expression may be beneficial for GBM, since knockout of VISTA renders mice high resistance against glioma tumours [34].

During aging, microglia are thought to become primed, dystrophic and senescent, leaving them less responsive and incapable of properly monitoring the CNS [101]; hence, microglia phenotypes associated with aging may contribute to the development of NDD such as AD and PD. Aged mouse cerebellar microglia exhibit reduced *VISTA* expression compared with microglia from younger mice (Fig. 3, Table 1). In humans, such a comparison is more difficult due to limited availability of post-mortem tissue from young individuals. However, *VISTA* expression is slightly increased in microglia from individuals > 50 years of age compared with < 50 years (Fig. 3, Table 1). This increase is much more pronounced in bulk tissue, which again supports the notion that other cell types may upregulate or induce VISTA upon deficits in CNS homeostasis.

## Therapeutic potential of VISTA in CNS disease

In oncology, immune checkpoint inhibitors (ICI) targeting NCR such as PD1 and CTLA4 have emerged as effective treatments that boost anti-tumour immunity. For autoimmunity, NCR-directed immunotherapy has more recently been explored in order to achieve inhibition of the immune system. ICI are systemic drugs affecting not only the periphery, but also the CNS. Therefore, ICI are used in the treatment of CNS-associated tumours, but these drugs can also have neurological side effects. VISTA is a more recently identified target for immunotherapy and blocking or activating VISTA has been proven effective in mouse models of cancer, inflammation and autoimmunity. The understanding of VISTA biology in the CNS is limited, and VISTA manipulation to enhance or mute its activity may offer novel therapeutic approaches for CNS diseases including GBM, NDD and MS.

## Targeting VISTA in autoimmunity and cancer

In mouse studies, VISTA has successfully been used as a target for immunotherapy in cancer and autoimmunity. The two main approaches that are used to block or enhance VISTA signalling are employing immunoenhancing anti-VISTA antibodies (antagonists) or immunosuppressive anti-VISTA antibodies (agonists), respectively. In addition to antibodies, small molecules or constructs can also be designed to target VISTA, leading to enhanced or suppressed immunity.

In multiple mouse models of cancer, an immunoenhancing anti-VISTA antibody (clone 13F3) leads to a reduction in tumour size and increased overall survival [102]. Blocking VISTA using immunoenhancing antibodies leads to increased infiltration of tumour-specific T cells, a decrease in MDSC numbers and suppressive capacity and a decrease in tumour-specific Tregs [102]. Using these immunoenhancing anti-VISTA antibodies in mouse models of autoimmunity including EAE [6] and murine lupus nephritis [11] exacerbates disease.

Immunosuppressive anti-VISTA antibodies reduce the severity of inflammatory disease in mice including autoimmunity. GvHD is prevented when targeting VISTA on donor T cells using an immunosuppressive anti-VISTA antibody [14]. Using this immunosuppressive antibody (clone MH5A or 8G8), disease severity of experimental asthma [41], lupus, hepatitis, psoriasis and arthritis [7] are reduced, and autoimmunity in systemic and discoid lupus erythematosus is alleviated [35].

In summary, VISTA can be used as a therapeutic target for both enhancing the immune response in case of cancer and inhibiting the immune response during inflammation and autoimmunity.

## Immunotherapy in the CNS

Immunotherapy using ICI is currently established as an effective treatment against several cancer types, and targeting immune checkpoints is more recently being explored as new treatment options for autoimmune diseases such as rheumatoid arthritis and MS. Studies mainly focus on the effects of immunotherapy on peripheral immunity; however, evidence strongly suggests that ICI affect the CNS as well.

Currently, there is no FDA-approved immunotherapy for GBM, but initial preclinical studies have yielded some encouraging results [3]. Since GBM tumour cells and infiltrating T cells express a range of NCR, targeting these checkpoints may boost the anti-tumour immunity. In CNS metastatic diseases, ICI targeting PD1 (pembrolizumab, nivolumab) and CTLA4 (ipilimumab) have been shown to slow down progression or reduce tumour size [4].

Immunotherapy may not only be beneficial in CNS-associated tumours, but also in NDD and MS. In AD, neuroinflammation is associated with increased hyperphosphorylated tau burden and microglia-mediated recruitment of peripheral immune cells can help in clearing amyloid β plaques [103]. Anti-PD1 antibody therapy facilitates clearance of amyloid β and improves cognitive performance in AD mice [104]. However, conflicting data exist that suggest there is no effect of anti-PD1 therapy in AD [28]. Currently, there are more than 10 FDA-approved immunomodulatory therapies for MS [105]. These drugs interfere with peripheral immune cell trafficking to the CNS, deplete subsets of immune cells or modulate immune signalling pathways; however, immune checkpoints are not used as a target for MS immunotherapy yet. Agonistic antibodies targeting NCR such as VISTA may enhance immune inhibition signals and could potentially present an effective treatment for MS.

Interestingly, ICI used in oncology have adverse effects on the CNS. Nivolumab treatment of melanoma induced spontaneous, reversible CNS demyelination in a patient [106]. ICI-treated patients are also more susceptible to developing other CNS diseases including paraneoplastic neurological symptoms, encephalitis, MS and hypophysitis, an inflammation of the pituitary gland [1]. In melanoma patients treated with ICI, these neurological adverse events occur in 1% (anti-CTLA4), 3% (anti-PD1) or 14% (anti-CTLA4 and anti-PD1) of the population [1]. These complications are likely caused by augmented immune responses leading to neurotoxicity. However, it is incompletely understood whether the ICI-associated CNS adverse effects and beneficial effects of ICI on CNS-associated tumours are mediated indirectly via infiltrating immune cells, directly by therapeutic antibodies gaining access into the CNS parenchyma at meaningful concentrations or by both. Since the BBB is compromised during MS and many CNS-associated tumours, it is highly likely that ICI can act on CNS-resident cells directly. Therefore, studying NCR expression and function in CNS-resident cells is crucial to developing ICI therapies for CNS diseases and to predict and mechanistically understand CNS adverse events.

## Approaches to modulate VISTA in CNS disease

Based on effectiveness of targeting VISTA in cancer and autoimmunity in mice and the use of ICI in CNS-associated tumours and NDD, it is conceivable that VISTA may offer a novel therapeutic target for treating CNS disease.

When targeting VISTA as a therapeutic strategy to treat NDD, CNS-associated tumours or MS, it is important to consider the complex expression dynamics and functions of VISTA. Using monoclonal antibodies against VISTA will not only target various peripheral immune cells (APC, neutrophils, T cells), but also microglia and CNS endothelial cells. Research has been focused on the function of VISTA in peripheral immune cell subsets, whereas there is no knowledge on the effects in the CNS of targeting VISTA using monoclonal antibodies.

Regarding the potential function of VISTA in the CNS, multiple functional outcomes of VISTA modulation are plausible. Targeting VISTA on endothelial cells may be a viable option to inhibit or enhance T cell activation during MS or NDD and cancer, respectively. In mice, VISTA knockout enhances anti-glioma responses in mice [34]. During MS, peripheral immune cell infiltration may be reduced upon enhancing VISTA signalling in endothelial cells. Microglia are APC and responsible for (re)activation of T cells in the CNS. Modulating VISTA on microglia may have similar effects as on endothelial cells. However, in microglia, VISTA may also be involved in phagocytosis, cytokine response and chemotaxis. These potential functions make it difficult to predict the outcome of modulating VISTA during peripheral and CNS disease. Using anti-VISTA antibodies systemically may affect microglia function unpredictably. It is therefore important to further dissect VISTA function in microglia in order to understand potential CNS responses to VISTA modulation. On the other hand, the large variety of functions that VISTA has in myeloid cells and potentially microglia may also open up treatment possibilities. For example, antibodies targeting different VISTA epitopes may have distinct functional consequences.

## Concluding remarks

VISTA represents an NCR with unique characteristics which is expressed predominantly by microglia in the CNS. Expression of VISTA is differentially regulated in ageing, neuroinflammation and multiple CNS diseases including neurodegeneration, stroke and cancer. Effective targeting of VISTA in cancer and autoimmunity opens wide possibilities to modulate VISTA a a therapeutic strategy in CNS disease. However, more knowledge on the functions of VISTA in the CNS and the effects of systemic VISTA modulation on the CNS is necessary to evaluate the therapeutic potential of targeting VISTA in CNS diseases. VISTA’s roles in microglia and the CNS are currently only beginning to be explored; hence, we have formulated remaining open questions in Box 1. Answering these questions will provide insights into the function of VISTA in microglia and in CNS disease, which will potentially yield novel therapeutic strategies and mechanistic insights into CNS homeostasis and disease.

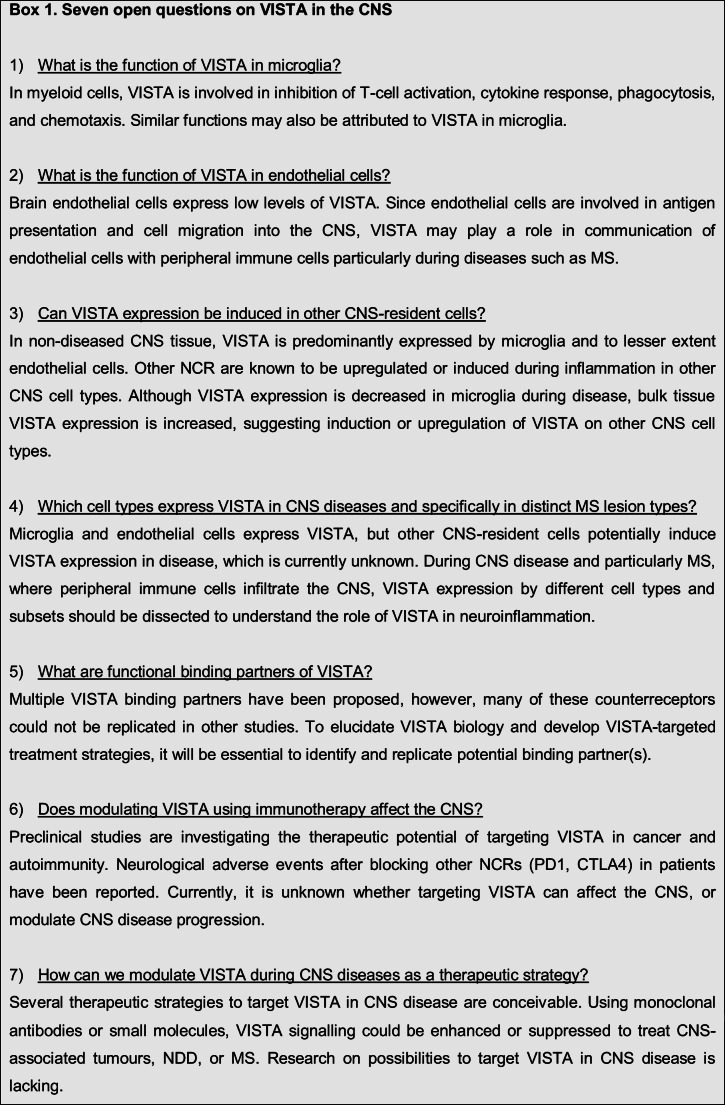



## Abbreviations

5XFAD: 5 familial Alzheimer’s disease mutations
A2AR: Adenosine A2A receptor
AD: Alzheimer’s disease
ALS: Amyotrophic lateral sclerosis
APC: Antigen-presenting cell
APOE: Apolipoprotein E
APP: Amyloid beta precursor protein
APP/PS1: APPswe/PS1dE9
ATAC-seq: Assay for transposase-accessible chromatin using sequencing
BBB: Blood-brain barrier
BM: Bone marrow
BTLA: B- and T-lymphocyte attenuator
CCL2: C-C chemokine ligand 2
CCR2: C-C chemokine receptor 2
CD: Cluster of differentiation
CDH23: Cadherin23
CFA: Complete Freund’s adjuvant
CNS: Central nervous system
CSF1(R): Colony stimulating factor 1 (receptor)
CTLA4: Cytotoxic T-lymphocyte-associated protein 4
Ctx: Cortex
CX3CR1: CX3C chemokine receptor 1
DAM: Disease-associated microglia
DC: Dendritic cell
DD1a: Death domain 1 alpha
EAE: Experimental autoimmune encephalomyelitis
ELISA: Enzyme-linked immunosorbent assay
FDA: Food and drug administration
FPKM: Fragments per kilobase million
GBM: Glioblastoma
GM: Grey matter
GvHD: Graft-versus-host-disease
HIF1a: Hypoxia-inducible factor 1 alpha
Hippo: Hippocampus
Homeo: Homeostatic (microglia)
ICI: Immune checkpoint inhibitor
IDO: Indoleamine 2,3-dioxygenase
IFNg: Interferon gamma
Ig: Immunoglobulin
IL: Interleukin
iNOS: Cytokine-inducible nitric oxide synthase
ITGAM: Integrin alpha M (= CD11B)
KO: Knockout
LCMV: Lymphocytic choriomeningitis virus
LogFC: Log2 fold change
LPS: Lipopolysaccharide
MCP1: Monocyte chemotactic protein 1 (= CCL2)
MDSC: Myeloid-derived suppressor cells
MFP2: Multifunctional protein 2
MOG: Myelin oligodendrocyte glycoprotein
mRNAseq: Messenger ribonucleic acid sequencing
MS: Multiple sclerosis
NA: Not available
NAWM: Normal-appearing white matter
NCR: Negative checkpoint regulator
NDC: Non-demented control
NDD: Neurodegenerative disease
NOX2: Nicotinamide adenine dinucleotide phosphate NADH oxidase isoform 2
OVA: Ovalbumin
Padj: Adjusted *P* value
PD(L)1: Programmed cell death (ligand) 1
PD-1H: PD1 homologue
PS1: Presinilin 1
PSGL1: P-selectin glycoprotein ligand 1
SOD1G93A: Superoxide dismutase 1 mutation G93A
SPF: Specific-pathogen free
TCR: T cell receptor
TIM3: T cell immunoglobulin domain and mucin domain 3
TLR: Toll-like receptor
tMCAO: Transient middle cerebral artery occlusion
TMEM119: Transmembrane protein 119
TNF: Tumour necrosis factor
TREM2: Triggering receptor expressed on myeloid cells 2
VISTA: V-type immunoglobulin domain-containing suppressor of T cell activation
VSIG: V-set and Ig domain-containing protein
WM: White matter
WT: Wild-type
